# Correction: Invasion inhibition in pancreatic cancer using the oral iron chelating agent deferasirox

**DOI:** 10.1186/s12885-023-11091-y

**Published:** 2023-06-23

**Authors:** Shogo Amano, Seiji Kaino, Shuhei Shinoda, Hirofumi Harima, Toshihiko Matsumoto, Koichi Fujisawa, Taro Takami, Naoki Yamamoto, Takahiro Yamasaki, Isao Sakaida

**Affiliations:** 1 Department of Gastroenterology and Hepatology, Yamaguchi University Graduate School of Medicine, 1-1-1 Minami-Kogushi, Ube, Yamaguchi 755-8505 Japan; 2 Department of Oncology and Laboratory Medicine, Graduate School of Medicine, Yamaguchi University, Ube, Yamaguchi Japan

**Correction: BMC Cancer 20, 681 (2020)**

**https://doi.org/10.1186/s12885-020-07167-8**

Following publication of the original article [1], the authors have identified an error, specifically the overlapping of the two images in Fig. 2a (24 h, DFX 10 μm and DFX 100 μM), which was pointed out to us by the editorial team. The corrected Fig. 2 is given in this correction article.

**Fig. 2:**
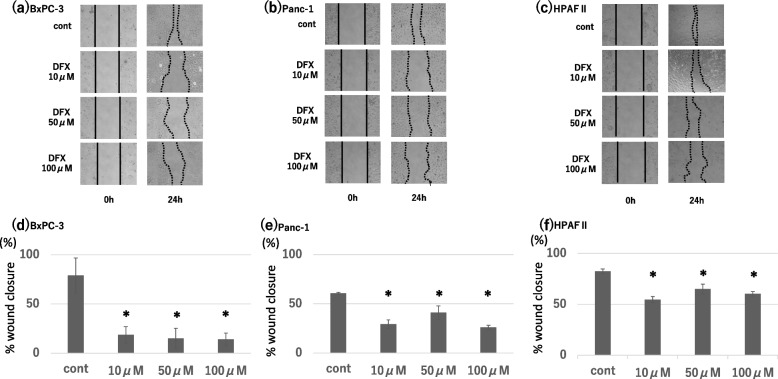
Effect of DFX on migratory ability of pancreatic cancer cells. Pancreatic cancer cell lines (BxPC-3, Panc-1, HPAFII) were treated with DFX (0, 10, 50, 100 μM) and incubated for 24 h. **a**-**c** Migrated cells were visualized via phase-contrast microscopy. **d**-**f** % wound closure was measured and the ratio to wound width at the start of the incubation was used as an index of cell migration, compared to control group (*n* = 3 each). Data are presented as mean ± SD. *P < 0.01 vs control
